# Manifestations of Age on Autophagy, Mitophagy and Lysosomes in Skeletal Muscle

**DOI:** 10.3390/cells10051054

**Published:** 2021-04-29

**Authors:** Matthew Triolo, David A. Hood

**Affiliations:** 1 Muscle Health Research Centre, York University, Toronto, ON M3J 1P3, Canada; trioloma@yorku.ca; 2 School of Kinesiology and Health Science, York University, Toronto, ON M3J 1P3, Canada

**Keywords:** skeletal muscle, aging, autophagy, mitophagy, lysosomes, sarcopenia

## Abstract

Sarcopenia is the loss of both muscle mass and function with age. Although the molecular underpinnings of sarcopenia are not fully understood, numerous pathways are implicated, including autophagy, in which defective cargo is selectively identified and degraded at the lysosome. The specific tagging and degradation of mitochondria is termed mitophagy, a process important for the maintenance of an organelle pool that functions efficiently in energy production and with relatively low reactive oxygen species production. Emerging data, yet insufficient, have implicated various steps in this pathway as potential contributors to the aging muscle atrophy phenotype. Included in this is the lysosome, the end-stage organelle possessing a host of proteolytic and degradative enzymes, and a function devoted to the hydrolysis and breakdown of defective molecular complexes and organelles. This review provides a summary of our current understanding of how the autophagy-lysosome system is regulated in aging muscle, highlighting specific areas where knowledge gaps exist. Characterization of the autophagy pathway with a particular focus on the lysosome will undoubtedly pave the way for the development of novel therapeutic strategies to combat age-related muscle loss.

## 1. Introduction

Skeletal muscle comprises approximately 40% of total body mass and is integral for locomotion and metabolic control [1]. Mitochondria, the organelles responsible for energy provision through the electron transport chain, make up approximately 2 to 7% of muscle cell volume [2,3]. These organelles also play an essential role within muscle through the production of reactive oxygen species (ROS), in Ca^2+^ handling, and in apoptotic signaling. Muscle cells display a high degree of adaptive plasticity, and as such, respond to the stimuli placed upon them, as commonly seen with endurance exercise and periods of disuse/inactivity. The lability of this tissue is controlled by a balance of organelle and protein biosynthesis, and their degradation. Autophagy is a major catabolic system within the cell, principally responsible for the selective degradation of long-lived proteins, protein aggregates and organelles via lysosomes [4]. Autophagic breakdown is essential for the maintenance of cell health through the removal of damaged or dysfunctional cellular components, such as mitochondria via mitophagy. Importantly, inhibition of autophagy in skeletal muscle elicits atrophy, perturbations in sarcomere structure, neuromuscular junction decay, and weakness [5,6,7,8,9].

With aging there is a progressive decline in muscle mass [1] and quality [10], commonly referred to as sarcopenia [10,11,12]. Concomitantly, mitochondrial content and function are reduced [13,14,15] which contributes to the metabolic decrements observed in aged skeletal muscle. In the elderly population, the effects of sarcopenia can be exacerbated by prolonged periods of inactivity related to injury or hospitalization [16]. Fortunately, exercise is capable of restoring muscle health following periods of disuse, and slowing age-related muscle wasting [14,17].

Further understanding of the molecular differences between young and old muscle in regard to the autophagy-lysosome system will guide the development of strategies to enhance skeletal muscle health with increasing age. Such an understanding will likely extend beyond age-related sarcopenia, as dystrophic, cachectic and lysosome storage disease muscles all commonly display altered autophagy, mitochondrial defects and muscle atrophy [18,19,20,21,22,23,24,25,26]. The goal of this review is to summarize the current understanding of the autophagy-lysosome system in aging muscle.

## 2. Regulation of Autophagy in Muscle

In its basic form, macroautophagy (herein referred to as autophagy) is the engulfment of materials in a double-membraned autophagosome, followed by their delivery to lysosomes for degradation. The process can be broken down into a series of steps, including initiation/activation, nucleation, elongation/maturation, transport, fusion with lysosomes, and degradation. These steps are regulated by a family of autophagy-related genes (*Atg*s), the function of which has been elucidated primarily in yeast and confirmed in mammalian cells/tissue. The steps and the key players have been reviewed extensively in the past [4,27], and we highlight these as a framework and describe the research that has been undertaken in the context of muscle physiology.

### 2.1. Overview of the Autophagy Pathway

#### 2.1.1. Transcriptional Control of the Autophagy Lysosome System

The transcription factor forkhead box O3 (FoxO3) and members of the MiTE/TFE family, particularly Tfeb and Tfe3, coordinate autophagy-lysosome gene expression pathways. FoxO3 is an integral transcriptional regulator of the muscle atrophy program through the ubiquitin-proteasome and autophagy systems [28,29,30,31]. FoxO3 also aids in the control of mitophagy through Bcl2 and adenovirus E1b 19-kDa-interacting protein 3 (Bnip3) and Bnip3-like (Nix) gene expression [31]. The importance of FoxO3 in muscle was shown in a series of seminal studies, whereby constitutively active FoxO3 promotes atrophy [28,30,31], whereas blunted FoxO3 activity prevents stimulus-induced atrophy of muscle fibers through perturbations in both proteasomal and autophagic pathways [28,32,33,34,35,36]. Tfeb and Tfe3 homodimerize and/or heterodimerize and interact with E-box and M-Box elements in promoters [37,38,39], and they regulate the expression of both lysosome and autophagy-related genes via their ability to activate the coordinated lysosome enhancement and regulation (CLEAR) network of genes [38,40,41,42,43].

#### 2.1.2. The Autophagy Pathway from Activation to Execution

Autophagy begins with the induction of a pre-autophagosomal structure, mediated by the Ulk1 complex [44,45,46,47,48,49]. Ulk1 activation is negatively regulated by the mammalian target of rapamycin complex 1 (mTORC1), and positively regulated by AMP-activated protein kinase (AMPK) [50,51,52,53], as discussed further below. Activated Ulk1 stimulates a class III PI3K Complex I (PI3KC3-C1), consisting of Vps15, Vps34, Atg14, Beclin1 and Ambra1 [54,55,56] to generate phosphatidylinositol-2-phosphate [PI(3)P], which is utilized in the nucleation of autophagosomal membranes [57,58]. Thus, Ulk1 activity is essential for the activation of autophagy in muscle, and knockout of Ulk1 perturbs autophagy flux [59,60].

Phagophore expansion occurs via two ubiquitin-like conjugation systems. The first creates the Atg12-Atg5-Atg16L1 complex, which aids in the second conjugation system, ultimately forming pro-microtubule-associated protein 1A/1V chain 3 (LC3) I, and then LC3-II. These processes are mediated by Atg3, Atg7, and Atg10 [58,61]. LC3-II and its related protein family members, γ-aminobutyric acid receptor-associated protein (Gabarap), and Golgi-associated ATPase enhancer of 16kDa (Gate16), embed in the autophagosomal membrane and act as receptors for cargo selection [62,63,64].

Cargo selection can be Ub-dependent or -independent. Both mechanisms utilize receptors that bind to digestible material directly, or via Ub-chains that are tethered to cargo [62,65,66]. Ultimately, these receptors interact with the autophagosome via an LC3-interacting region (LIR) [67,68]. Autophagosomes are then transported across microtubule tracts to lysosomes [58,69,70], where the membranes of these two compartments fuse, forming an autolysosome. This is mediated by Ras-related protein 7 (Rab7), Snare proteins and lysosomal associated membrane proteins (Lamps) [69].

#### 2.1.3. Important Considerations in Autophagic Measurements

Various studies investigating autophagy regulation highlight alterations in autophagic markers and make inferences about the activation or repression of the autophagy pathway. Although such studies are informative, they do not accurately encapsulate the dynamics of the autophagic process. Critical to this analysis is the ability to assess flux through the pathway, given its dynamic nature. In the presence of inhibitors, such as the microtubule destabilizer colchicine, which dissociates autophagosome formation from transport to the lysosomes for terminal degradation, or bafilomycin A, which inhibits autophagosome-lysosome fusion, a comparison of autophagosomal proteins (i.e., LC3-II or p62) between drug- and vehicle-treated conditions is a reliable measure of autophagy flux [71]. However, such methodologies come with limitations. For example, these assays fail to capture the role of the lysosomes in autophagic resolution, and may also create a backlog of autophagic substrates, thereby creating intracellular stress. As such, these agents may alter the phenotype of the tissue, and this can impose a confounding variable. Thus, other assays have also been developed which may be feasible to apply to the muscle biology field, including the use of fluorescent-labelled markers and pH-sensitive probes for lysosome function (i.e., Mitotimer or mtKeima) [72]. These methods are useful in pre-clinical animal models or in cell culture, but they cannot be applied to the assessment of autophagy in human muscle, where the reliance on autophagy markers which correlate with flux is required. This technicality currently limits our understanding of autophagy in human skeletal muscle physiology.

### 2.2. Upstream Regulation of the Autophagy Lysosome System

#### 2.2.1. Coordinated Activation of the Autophagy Lysosome System by Nutrient Availability

**Figure 1:**
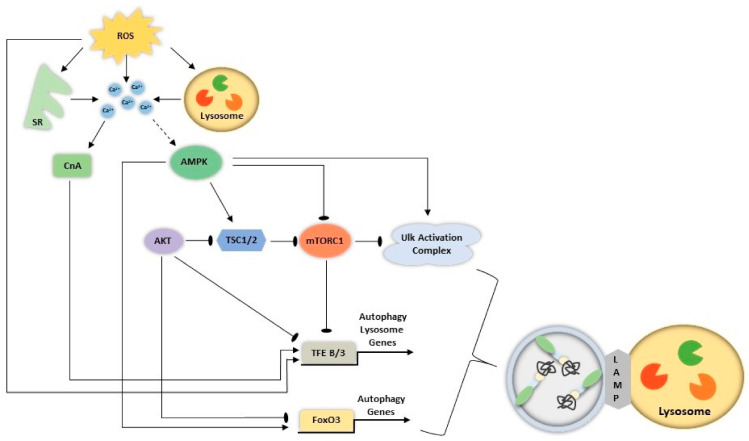
Proposed coordination of pathways that regulate the autophagy-lysosome System (ALS). ROS and cytosolic calcium ([Ca^2+^]_IC_) act as upstream signaling mechanisms that converge on the gene expression and protein activation of the ALS. AMPK is activated directly via compromised energy status (increase AMP:ADP), and indirectly through [Ca^2+^]_IC_ via CaMKII (not shown). Elevated [Ca^2+^]_IC_ may occur due to the damaging effects of elevated ROS, from mitochondria or other sources, on the sarcoplasmic reticulum and lysosomes. AMPK synchronously activates FoxO3, Tfeb and Tfe3 (Tfeb/3) through (1) direct phosphorylation of FoxO3, (2) activation of TSC1/2 and (3) inhibition of mTORC1. Simultaneously, AMPK activates autophagy induction through the Ulk1 complex and by alleviating mTORC1 inhibition on this complex. In a similar cellular milieu, FoxO3 repression is alleviated via AKT inhibition. Increases in [Ca^2+^]_IC_ activate the phosphatase CnA, which activates Tfeb/3. ROS also activate Tfeb/3 through oxidation, an added layer of coordination in this autophagy-lysosome system.

Autophagy-lysosome related genetic programs require sensing of the cellular milieu to coordinate the expression of essential gene-products. As such, FoxO3, Tfeb and Tfe3 undergo a series of post-translational modifications which dictate their nuclear localization and activity. Further, the Ulk1-complex, at the early stage of autophagy induction, is also tightly integrated with the signaling mechanisms that regulate these transcription factors, ultimately leading to a coordinated ALS activation. These pathways are summarized below and depicted in Figure 1.

Under fed conditions, FoxO3, Tfeb and Tfe3 localization is primarily cytosolic due to repression mediated by active AKT [73,74]. mTORC1, positioned downstream, and localized to the lysosome [52], phosphorylates Tfeb on Ser^211^ [75] and Tfe3 on Ser^321^ [41]. This promotes their interaction with 14-3-3 in the cytosol, rendering them inactive [41,75,76,77]. mTORC1 also inhibits Ulk1 through direct interaction and phosphorylation [78]. In this cellular milieu, AMPK activity is low, and thus its impact on activating FoxO3 and Ulk1-complex through phosphorylation is minimal [73,79,80,81]. These conditions promote a coordinated suppression of the autophagy-lysosome program.

In contrast, under energy stress, when AMPK is active, FoxO3 [73,79,80] and Ulk1 [81] activity are enhanced through direct phosphorylation. AMPK phosphorylates and inhibits mTORC1 [82] and the mTORC1 inhibitor Tsc1/2 [83] which will ultimately alleviate mTORC1′s repression on Ulk1, and Tfeb/Tfe3 to promote their nuclear localization. In this way, AMPK ultimately coordinates the activation and induction of the autophagy-lysosome system.

The importance of these signaling pathways has been shown, whereby Tsc1 knockout blocks autophagy induction and promotes muscle atrophy [84]. However, this effect can be prevented by the mTOR inhibitor rapamycin [84], highlighting the importance of mTORC1 in repressing autophagic induction. Further, AMPK knockout leads to an attenuation of atrophy [85,86], likely due to a failure to induce autophagy [87] and to promote protein degradation. Thus, this regulatory network, including both mTORC1 and AMPK are essential for the maintenance of muscle mass.

#### 2.2.2. Other Mechanisms Coordinating Activation of the Autophagy Lysosome System

Other integral regulators of this coordinated response include reactive oxygen species (ROS) and Ca^2+^ [88,89,90,91,92]. Specifically, ROS activate AMPK [93] thereby enhancing FoxO3 and Ulk1 activity. ROS also inhibit AKT signaling [91,94] and ultimately the mTORC1 pathway [95], thereby alleviating its suppression on Ulk1 and FoxO3, further promoting autophagic induction. ROS can also potentiate CLEAR network expression via direct oxidation on Cys^212^ and Cys^322^ of Tfeb and Tfe3, respectively, which trigger their nuclear localization in muscle cells [95]. Finally, through oxidation of lysosomal transient receptor potential mucolipin 1 (Trpml1) on lysosomes [96] as well as Ryanodine Receptors (RyR) on the sarcoplasmic reticulum [97], ROS can promote the elevation of intracellular calcium ([Ca^2+^]_IC_) [89]. [Ca^2+^]_IC_ may induce autophagy via the activation of CAMKK*B* and subsequently AMPK, which has implications for Ulk1 and FoxO3 phosphorylation, as described above [98]. Furthermore, elevations in cytosolic Ca^2+^ facilitate nuclear localization of Tfeb [99] and Tfe3 [100] via the phosphatase calcineurin (CnA), which dephosphorylates these proteins.

### 2.3. Autophagy in Aging Muscle

#### 2.3.1. Upstream Regulation of Autophagy in Aging Muscle

A hallmark characteristic of sarcopenia is muscular atrophy, the result of a progressive loss of muscle protein over time. This could be achieved by an imbalance between muscle protein synthesis and the pathways that mediate protein degradation, including the ubiquitin proteasome pathway, apoptosis, and autophagy. Much remains to be elucidated regarding the signaling toward, and the activity of, autophagy-lysosome regulatory proteins. For example, there is evidence of upregulated FoxO3 [101,102,103] and Tfeb [102] content in aged muscle, supporting elevated expression of the ubiquitin-proteosome system and autophagy genes in old muscle. However, there are no clear indications of whether AKT-mediated FoxO3 phosphorylation [104,105] and localization [104,106] are enhanced or reduced with age. This may be explained, in part, by the relatively inconsistent measures of AKT activity that exist in the literature [104,106,107,108,109]. Further, deacetylation of FoxO3 by histone deacetylase 1 (HDAC1) [73,110,111] and co-activation by CARM1 [32,33] enhance FoxO3 function, and are implicated in disuse/denervation atrophy. Both mechanisms have not been investigated in aged muscle. On the other hand, AMPK signaling is lower in aged individuals [112,113], and although the ramifications of this for FoxO3 activity is yet to be uncovered, it has been shown that, the muscle of aged humans and rats contain lower basal AMPK-activating-Ulk1 phosphorylation [59]. Furthermore, mTORC1 activity is sustained in aged muscle [114,115,116], corresponding to increases in inhibitory Ulk1 phosphorylation [107,114,117]. This mTORC1-mediated inhibition may mitigate the extent of atrophy in aging muscle. However, no reports have investigated Tfeb or Tfe3 activity or localization in aged muscle. The lack of data on these signaling pathways suggest that more work is necessary to understand the influence of the autophagy-lysosome system in mediating the atrophy evident in aging muscle.

Both ROS [118,119,120] and [Ca^2+^]_IC_ [97,118,121,122] are elevated in aged skeletal muscle, which may promote both autophagy-lysosome related gene expression and autophagic induction. In fact, scavenging of ROS via antioxidant and genetic models, in muscle disuse [94,123] and denervation [124,125,126] suppresses autophagic pathways and muscle loss, which may be true in aging muscle. Similarly, it would be essential to understand how age-related [Ca^2+^]_IC_ elevations impact the autophagy lysosome system, as the literature has been primarily focused on Calpain activity in response to Ca^2+^ overload. Cumulatively, these limited data also point to the requirement of assessing the necessity of ROS- and Ca^2+^-induced autophagy signaling in aging muscle.

#### 2.3.2. Autophagy Flux in Aging Skeletal Muscle

There are a variety of reports that support both enhanced, as well as reduced, autophagy in aged skeletal muscle, based on assessment of protein markers. Evidence of normally functioning autophagosome formation, with impairments in the processing and degradation of autophagosomes was observed based on accumulations in Beclin-1, LC3-I and LC3-II, without a change in upstream autophagy markers Atg7 and 9 or *Lc3* transcript [127]. Our laboratory and others have similarly reported elevations in LC3-II, its processing protein Atg-7, LC3-II/I, and p62 [103,128,129,130,131]. In humans, Fry et al., measured elevations in autophagic proteins Atg7 and Beclin1 without changes in LC3-II/I, a result that is suggestive of perturbed autophagosomal breakdown with age [132].

In contrast, others have documented no change in Beclin1 or LC3-II/I in humans [133], and LC3-II/I or p62 protein expression in aged mice [107], findings that were interpreted as unaltered autophagy with age. These changes in autophagy execution may be fiber-type specific as some investigations have found that protein markers such as Atg5, LC3 and p62 are maintained in slow, but not fast muscle [134]. This fiber type difference is relevant in young, healthy muscle, whereby oxidative muscle has greater autophagy flux than predominantly fast muscle [135]. This fiber-type specific response requires further investigation.

Inconsistencies in these investigations may be explained by the “snapshots” measures of autophagosomal transcript and protein levels used to determine autophagy, which fail to capture the dynamic nature of the pathway. Recently, two studies utilized colchicine to assess autophagy flux in aged Fisher344BN rats. Baehr et al., measured an accumulation of p62 and LC3-II in the soleus and tibialis anterior muscles of 29mo rats [136], suggesting diminished autophagic degradation. However, autophagy flux was enhanced in the aged cohort [136]. Similarly, we have recently reported elevation in autophagy flux in 36mo rats [102]. Thus, it appears that autophagy flux in muscle is elevated with age, at least up until the point of cargo delivery to the lysosome.

#### 2.3.3. Ramifications on Aging Skeletal Muscle

Although these basal changes in autophagy are important, understanding the extent to which muscle adapts to stimuli placed upon it is critical. In response to starvation, aged muscle fails to elicit the AKT and mTORC1 inhibition that is typical of young muscle [114]. Furthermore, there is also evidence of blunted stimulus-induced AMPK [137,138,139] and mTORC1 [140] activation, supported by lower starvation-induced Ulk1 activation [114]. Interestingly, caloric restriction [127], endurance exercise training [127], and chronic muscle activity [102] do improve autophagic degradation in aged muscle, however, the response is curtailed compared to a younger cohort. These data provide evidence that senescent muscle has a perturbed autophagy program. This has widespread ramifications for the ability of aged muscle to adapt to stimuli such as exercise and caloric restriction, which impact muscle quality.

## 3. Regulation of Mitophagy in Muscle

**Figure 2:**
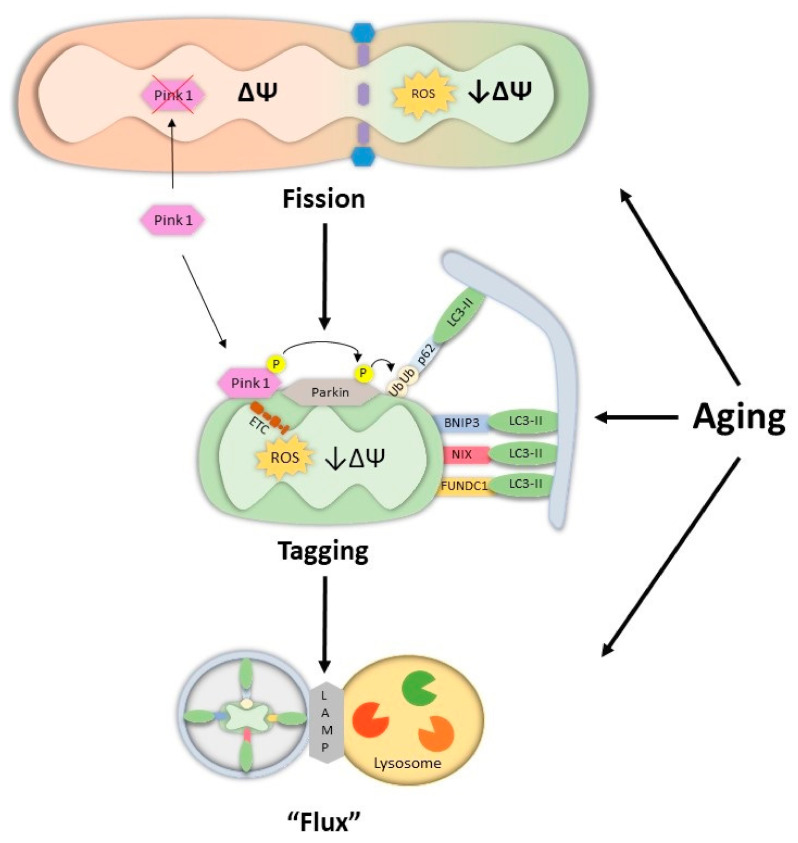
The impact of age on mitophagy in skeletal muscle. When a portion of the mitochondrial reticulum becomes dysfunctional, with a low membrane potential (Ψ) and elevated ROS production, it will undergo fission, mediated by Drp1 and Fis1, depicted as green mitochondria. The fragmented mitochondrion will enter the mitophagy pathway. Pink1, which is normally imported and degraded in the mitochondria, will accumulate on the outer mitochondrial membrane (OMM), and will ultimately recruit and activate the E3-Ligase Parkin. Parkin subsequently poly-ubiquitinates OMM proteins which bind to adapt proteins such as p62. p62 will tether autophagosomal proteins, such as LC3-II. In Ub-independent mitophagy, receptors such as BNIP3, NIX and FUNDC1, found on the OMM, will anchor the mitochondria directly to autophagosomal proteins. Ultimately the autophagosome-bound mitochondria will be shuttled to lysosomes where they will be degraded. Aging muscle exhibits increased mitochondrial fission, autophagy tagging, and flux, to the point of the lysosomes. Evidence, somewhat limited, exists that point to lysosomal dysfunction with age.

Mitochondrial health within muscle depends on organellar turnover that is a product of the balance between the synthesis of new, healthy organelles via biogenesis, and the recycling of damaged or dysfunctional mitochondria through mitophagy. This ensures the sustenance of a mitochondrial reticulum which is well coupled, thereby producing ATP efficiently and generating a level of ROS sufficient to signal events that maintain homeostasis. When segmental mitochondrial dysfunction occurs, evident form varying degrees of respiratory impairment, elevated ROS production, or loss of membrane potential, these organelles are digested at the lysosomes via the mitophagy pathway, as depicted in Figure 2, and as descried below. If balanced with a concurrent change in mitochondrial biogenesis, this turnover maintains the metabolic capacity of the muscle [13,14]. However, if these processes become unbalanced, the overall health of the mitochondrial pool will be disrupted, a phenomenon which is commonly observed in both aging and muscle disuse [13,14,119,120].

### 3.1. Mitochondrial Dynamics in Muscle—Implications for Mitophagy

Skeletal muscle mitochondria exist in a reticular network which optimizes the metabolic capabilities of the tissue [2,141,142]. This network is maintained through the opposing processes of fusion and fission. As recently reviewed [143], mitochondrial dynamics are regulated via the pro-fusion proteins, mitofusin 1/2 (Mfn1/2) and optic atrophy protein 1 (Opa1), as well as the pro-fission dynamic-related protein 1 (Drp1) and mitochondrial fission protein 1(Fis1). When a portion of the network becomes impaired it is sequestered through mitochondrial fission, which primes this organelle segment for its ultimate degradative fate via the mitophagy pathway [144,145].

The vitality of these fusion and fission regulatory proteins has been revealed using genetic models. For example, knockdown of the pro-fission proteins Fis1 and Drp1 propagates muscle atrophy [146,147,148], promotes hyperfused mitochondria with impaired function [149] and perturbs mitophagy [148,149]. These data suggest that fission is required to maintain muscle organellar health via the provision of dysfunctional mitochondria through mitophagy pathway. With regard to fusion, Mfn2 [129,150] and Opa1 [151] deletion promotes mitochondrial fragmentation, excessive ROS production and muscle atrophy. In combination, these results suggest that a balance of fusion and fission is required to maintain a healthy mitochondrial pool, which has implications for muscle health with age.

#### Mitochondrial Dynamics in Aged Muscle

A number of studies have reported a greater abundance of fission relative to fusion proteins in aged muscle [113,129,130,131,152,153], although some inconsistent findings have been observed [154,155]. Elongated mitochondria in aged muscle have been noted, a finding the authors attributed to intrinsic mitochondrial defects that promote fusion as a compensatory mechanism [155]. Variability in the results may be explained by the aging model employed, species differences, or the degree of aging examined. However, as mitochondrial dysfunction is apparent in aged muscle, and as fission precedes mitophagy, it is not surprising that there is evidence of fragmented mitochondria in aged muscle, which may prime this organellar pool for mitophagy.

### 3.2. The Process of Mitophagy

#### 3.2.1. Pink1-Parkin Mediated Mitophagy

The most well understood mitophagy system is termed ubiquitin-dependent mitophagy, which is controlled by the serine/threonine kinase, PTEN- induced putative kinase 1 (Pink1), and the E3-ubiquitin ligases Parkin. Pink1 is normally maintained at low levels within the cytosol under homeostatic conditions, as Pink1 is efficiently imported into functional mitochondria, processed by the mitochondrial protein peptidase (MPP) and subsequently degraded by the intramembrane protease presenilin-associated rhomboid-like protease (PARL) [156,157,158], or by Lon protease [159]. Following mitochondrial stress, particularly an accumulation of damaged intraorganellar proteins [160] or a loss of membrane potential [156,161,162,163], protein import is hindered, and Pink1 accumulates on the outer mitochondrial membrane (OMM). Here, Pink1 undergoes autophosphorylation and activation [164] to phosphorylate both Parkin [161,162,163,165] and ubiquitin [166,167,168,169]. The result is polyubiquitination of OMM proteins such as VDAC, Mfn1/2, mitochondrial translocase complex proteins (TOMs) and ubiquitin, which will feedforward to recruit more Parkin and ubiquitin to the membrane [161,162,163,170,171]. Ultimately, these ubiquitin chains act as a scaffold for adapter proteins such as p62 [172,173] optineurin [173,174] and Ndp52 [173], which interact with the autophagosomal proteins leading to organelle engulfment and subsequent degradation. An essential Parkin-regulated process is the ubiquitination and degradation of the fusion protein Mfn1/2, ensuring that re-fusion does not occur, and allowing for mitophagy to take place effectively [175].

The importance of Pink1/Parkin-mediated mitophagy in skeletal muscle has been demonstrated in lower organisms [176,177], cultured myotubes [178,179] and mice [180,181]. Specifically, Parkin knockout promotes muscle degeneration and cell death in drosophila [176], as well as insulin-insensitivity [178] and susceptibility to mitochondrial toxins [179] in myotubes. In the muscle of Parkin-null mice, there is evidence of mitochondrial fragmentation [182], impaired mitochondrial respiration [180,181,182] and reduced mitophagy flux [180,181]. Interestingly, Parkin-null mice fail to moderate mitophagy in response to acute exercise [180] and endurance training [181]. Parkin overexpression in mice prevents both aging- and sepsis-induced loss of muscle mass and strength [183,184] and extents lifespan through reduced proteotoxicity in drosophila [177]. Cumulatively, these reports highlight the importance of the Pink1-Parkin-mediated mitophagy system in skeletal muscle.

#### 3.2.2. Receptor Mediated Mitophagy

Ubiquitin-independent mitophagy, also referred to as the receptor-mediated mitophagy system, operates more directly than the Parkin-driven pathway, and it is unclear if these mechanisms occur simultaneously or independently. Mitophagy receptors that have been characterized include Bnip3 [185,186,187], Bnip3-like protein (Nix) [188,189,190] and Fun14 domain containing protein (Fundc1) [191,192], which are normally localized to mitochondria [191,193,194,195]. Upon phosphorylation, these receptors interact with phagophore proteins such as LC3 and Gabarap [189,191,196]. In the early stages of mitophagy, Bnip/Nix also promote the mitochondrial localization of Drp1, which would enhance fission and aid in Parkin recruitment, leading to mitophagy [197,198,199].

The importance of these mitophagy mechanisms in muscle has recently been demonstrated. Knockout of Bnip3 in muscle cells prevented their differentiation, likely a result of an accumulation of dysfunctional mitochondria [200]. Similarly, knockout of both Nix and Fundc1 in cardiac progenitor cells led to cell death [201]. Further, skeletal muscle deletion of Fundc1 impaired mitochondrial function and exercise capacity, and this protein was required for both basal as well as exercise and contraction-induced mitophagy [202,203]. In contrast, the overexpression of the Bnip3 and Nix induced muscle atrophy [146], providing evidence that mitochondrial degradation is fine-tuned to the maintenance of muscle mass.

### 3.3. Mitophagy in Aging Skeletal Muscle

#### 3.3.1. Mitophagy Targeting in Aged Muscle

As mitochondrial fragmentation and dysfunction are evident in senescent muscle, one would suspect that mitophagic signaling would be enhanced to remove these dysfunctional organelles. A variety of studies have reported the enhanced expression of the mitophagy proteins Bnip3, Nix, Pink1, and Parkin [102,129,180,204,205], but some studies have measured either no change and/or reduced levels of Bnip3 and Nix [130,133,206] in aged muscle. The phosphorylation status of these proteins would provide a clear indication if these mitophagy pathways are active, but this has not yet been explored in the context of aging muscle. We and others have reported decreases in Parkin protein [184] and mitochondrial Parkin [180] in 18 month old mice. In contrast, older rodents (i.e., 24mo mice and 35mo rats) exhibit elevations in Pink1, Parkin and Ubiquitin [130,131], suggesting an age-specific Pink1-Parkin mitophagic response.

#### 3.3.2. Mitophagy Flux in Aged Muscle

Supportive of enhance targeting, our laboratory has reported elevated autophagosomal tethering to mitochondria in aged muscle, as measured by increased LC3-II [131] and p62 [102,131] in these isolated organelles. These data may suggest either (1) an enhanced rate of organellar targeting and degradation or (2) perturbed efficiency of mitophagic breakdown. Using colchicine, we have observed increased mitophagy flux in aged mouse [180] and rat [102] muscle, which occurs alongside deficits in mitochondrial biogenesis [13,14,15]. Although enhanced flux would implicate the accelerated removal of damaged mitochondria, the remaining presence of impaired organelles indicates that the rate may not be great enough to eliminate all dysfunctional cellular components, including mitochondria. This remaining organellar pool will not only have less metabolic capacity, but also contribute to oxidative damage within the muscle that can propagate further dysfunction. Thus, restoring mitophagy in aged muscle may hold therapeutic benefit in slowing age-related sarcopenia.

Aging is associated with both neuromuscular denervation and reduced physical activity. Since it is known that denervation [124,126,131,207,208,209,210,211,212,213] and muscle disuse [214,215,216] independently impact mitophagic pathways, work is required to determine the independent nature of these stimuli on mitophagy. Furthermore, acute endurance exercise acts as a stimulus that promotes mitophagy in young muscle, used to prune the mitochondrial pool and create a healthier tissue [13]. However, there is evidence for impaired acute exercise-induced mitophagy in aged muscle [180]. Further both the chronic contractile activity induced reduction of mitophagy flux [102] and the denervation-induced mitophagic upregulation [131] are impaired in aged muscle. Thus, it is becoming clear that aging alters the mitophagic phenotype of muscle, and the lack of appropriate stimulus-induced alterations in mitophagy may limit positive adaptations in the maintenance of a healthy organelle population.

## 4. The Lysosomes and Aging Muscle

Lysosomes are highly acidic organelles that contain an abundance of enzymes to aid in the degradation of autophagic substrates. Typically thought of as the digestive organelle, the literature has implicated the lysosome as a significant component in the monitoring and response to changes in the cellular milieu, such as nutrient availability [217,218]. Lysosomes are also an important intermediate in organellar communication, for example, between the nucleus and mitochondria [219,220].

Lysosomes are created through the integration of gene expression and protein trafficking with the endocytic pathway, processes that are not well characterized in skeletal muscle. Indeed, the role and importance of lysosomes have been elucidated in many cell types, but limited work has been conducted in the skeletal muscle field. Yet, the value of these organelles in muscle is clear when viewed in the context of Lysosomal Storage Diseases (LSDs). For example, Pompe [5,221,222,223,224,225,226] and Danon Disease [227,228,229,230,231,232] are both associated with an accumulation of non-digested autophagosomes and enlarged lysosomes, manifesting in diminished muscle mass and function.

### 4.1. Lysosomal Perturbations in Aging Muscle

Within aging tissue, there is a progressive increase in lipofuscin, commonly referred to as “the age pigment”, indicative of dysfunctional lysosomal clearance [233,234]. Skeletal muscle is not exempt from this phenomenon, as lipofuscin granules are visible in both rodent [131] and human [235] muscle. We recently reported increases in the lysosomal proteins Lamp2 and Cathepsin D in the tibialis anterior of aged rats, which may be due to the observed increase in Tfeb [102] and its function in controlling lysosomal synthesis. Others have reported similar findings in the extensor digitorum longus muscle of mice [134], although transcript levels may be reduced [127,236]. Presently there is a lack of investigation into lysosomal function in muscle, despite occasional measures of gene expression and enzyme activity. Disturbances in lysosome function in aged muscle would have widescale implications for autophagy and mitophagy. This could explain why dysfunctional mitochondria and oxidized proteins accumulate in the muscle of aged animals and humans. As the “Lysosomal Theory of Aging” would suggest, this blunted auto- and mitophagic capacity may in fact promote further damage within the tissue, which could potentiate myonuclear loss through apoptosis mediated by both mitochondria through the release of cytochrome c, and lysosomes via the release of cathepsins. In line with this, it is established that mitochondrially driven apoptosis is enhanced in aged skeletal muscle [237,238,239], but it remains unclear if lysosome-mediated apoptosis is a contributing factor in determining the degree of sarcopenia.

### 4.2. Future Work of Lysosomes in Aging Muscle

As noted above, there is virtually nothing known about lysosomal function in aging muscle. In other cell types, dysfunction is characterized by a loss in acidification due to membrane instability, organelle swelling, and the release of active cathepsins which can ultimately lead to cell death [240]. Thus, it seems wise to dedicate future investigations toward these types of analyses in young and old muscle. This is because the importance of enhancing lysosomal health is established in various experimental models. For example, the overexpression of Tfeb is able to reduce pathophysiology through the transcriptional induction of components of the autophagy-lysosome system in neuropathies [241,242,243]. Similarly, a direct Tfeb activator, curcumin analog C1, is able to prevent the pathology associated with Alzheimer’s disease [244]. In muscle, AAV-mediated TFEB overexpression attenuated the severe pathology evident in a murine model of Pompe Disease [221]. As aging muscle displays lysosome impairments, Tfeb activation and subsequent restoration of lysosome integrity may ultimately restore muscle health, at least in part. In addition, an assessment of the impact of ROS and Ca^2+^ on the lysosomes in aged muscle is warranted. This is because both are elevated in aging muscle and can impact Tfeb and Tfe3, and ultimately lysosomal biogenesis, as discussed above.

## 5. Conclusions

In recent years significant advances have been made to uncover the mechanisms that underly age-related sarcopenia. Although there are multiple mechanisms involved, this phenotypic change is mediated by alterations in protein and organelle homeostasis. One integral mediator of protein turnover is the activity of the autophagy-lysosomes system, and its interaction with mitochondrial turnover. Mitochondria are a predominant source of oxidative stress and are degraded via this pathway. Insufficient autophagic clearance can lead to a build-up of poorly functioning organelles that perpetuate intracellular damage. In aged muscle there is evidence of (1) oxidative damage, (2) mitochondrial dysfunction and (3) lysosome impairments. Thus, the activity of the complete autophagy-lysosome system, from organelle-tagging to destruction within lysosomes, may become insufficient with age. Moving forward, research has identified the key proteins within the autophagy pathway upstream of the lysosome with age, but current knowledge of lysosome function is lacking as it relates to age-related muscle atrophy. Once this is better appreciated, recognition may follow on how enhancing lysosome function pharmacologically can slow potentially slow the progress of sarcopenia.

## Abbreviations

AMP activated protein kinase	AMPK
Autophagy-related genes	Atg
Bcl2/adenovirus E1B 19-kDa protein-interacting protein 3	Bnip3
BNIP3-like protein	Nix
Calcium/calmodulin-dependent protein kinase kinase B	CAMKK*B*
Calcineurin	CnA
Class III PI3K Complex I	PI3KC3-C1
Coactivator Associated Arginine Methyltransferase 1	CARM1
Coordinated lysosomal enhancement and regulation	CLEAR
Dynamic-related protein 1	Drp1
Fission protein 1	Fis1
Forkhead box O3	FoxO3
Fun14 domain containing protein	Fundc1
γ-aminobutyric acid receptor-associated protein	Gabarap
Golgi-associated ATPase enhancer of 16kDa	Gate16
Histone Deacetylase 1	HDAC1
LC3-II interacting region	LIR
Lysosomal-associated membrane protein	Lamp
Lysosomal storage disorder	LSD
Lysosome transient receptor potential mucolipin 1	Trpml1
Mammalian target of rapamycin	mTOR
Mitochondrial protein peptidase	MPP
Mitofusin 1/2	Mfn1/2
Optic atrophy protein 1	Opa1
Outer mitochondrial membrane	OMM
Phosphatidylethanolamine	PE
Presenilin-associated rhomboid-like protease	PARL
PTEN induced putative kinase	Pink1
Ras-related protein	Rab
Reactive Oxygen Species	ROS
Ryanodine Receptors	RyR
Sequestosome 1	Sqstm1/p62
*Trans*-golgi network	TGN
Translocase of the outer membrane	TOM
Tuberous Sclerosis Complex 1/2	Tsc1/2
Transcription factor EB	Tfeb
Transcription factor E3	Tfe3
Ubiquitin	Ub
